# Contrasting La Crosse Virus Lineage III Vector Competency in Two Geographical Populations of *Aedes triseriatus* and *Aedes albopictus* Mosquitoes

**DOI:** 10.3390/biology14121771

**Published:** 2025-12-11

**Authors:** Lindsey R. Faw, Philip M. Armstrong, Sally L. Paulson, Gillian Eastwood

**Affiliations:** 1 Department of Entomology, College of Agriculture and Life Sciences, Virginia Polytechnic Institute and State University, Blacksburg, VA 24061, USA; lrfaw@vt.edu (L.R.F.); spaulson@vt.edu (S.L.P.); 2 Center for Emerging, Zoonotic, and Arthropod-Borne Pathogens (CeZAP), Virginia Polytechnic Institute and State University, Blacksburg, VA 24061, USA; 3 Connecticut Agricultural Experiment Station (CAES), New Haven, CT 06511, USA; philip.armstrong@ct.gov; 4 The Global Change Center at VT, Virginia Polytechnic Institute and State University, Blacksburg, VA 24061, USA

**Keywords:** La Crosse virus, mosquito, *Aedes*, arbovirus, novel, viral lineage, transmission, competency, emergence

## Abstract

La Crosse virus (LACV) is a mosquito-borne virus (family *Peribunyaviridae*) that can result in severe human infection in children under sixteen. Historically, LACV comprised two lineages, predominantly in the Midwest and Appalachian regions of the US. In 2005, a virus of a third lineage was detected in the Northeast; however, this and subsequently isolated strains of lineage III have not, to date, been implicated in human disease. One hypothesis for this discrepancy is that vector mosquitoes have a reduced vector competency for LACV lineage III, thus preventing horizontal transmission and clinical cases. Here, we utilized two mosquito species, *Aedes triseriatus*, the native vector, and *Aedes albopictus*, an invasive potential vector, each from both a historic LACV range (Virginia) and from the region of lineage III (Connecticut). Utilizing oral feeding and intrathoracic inoculation methods of viral exposure, rates of LACV infection, dissemination, and transmission (proxied via salivary secretion) and capability for vertical transmission (proxied via virus-positive ovaries) were determined by harvesting mosquito bodies, legs, saliva, and ovaries, respectively. Overall, we did not detect consistent differences in transmission between any lineage, species, or state of origin, at day 14 post-infection. However, we highlight the transmission potential of LACV lineage III in all mosquito populations tested here, representing the first evidence of lineage III competency in *Aedes triseriatus* and *Aedes albopictus*, indicating the potential for human disease. We thus suggest that the absence of human cases of LACV lineage III is not modulated by a lack of vector competency in mosquitoes.

## Simple Summary

La Crosse virus (LACV) is a mosquito-transmitted virus in the United States that can cause a serious illness in children, with neurological symptoms such as seizures and swelling of the meninges and brain. Historically, there have only been two genetically distinct lineages of LACV that cause disease and correlate with geographic regions. In the Midwest and Appalachia, lineage I accounts for most of the LACV cases; lineage II is present in the South. In recent years, a new lineage, lineage III, has been detected in mosquitoes in the Northeast region, yet there have so far been no human cases attributed to lineage III. One possible reason for this is reduced vector competency (the ability of a mosquito to transmit the virus) for these novel strains of virus. We explored the vector competency of both the known LACV vector (*Aedes triseriatus*) and a suspected invasive vector (*Aedes albopictus*), with populations from Connecticut (lineage III area) and Virginia (a lineage I area). Using infection outcomes measured at 14 days post-infection, we demonstrated that both mosquito species are competent vectors for the virus, the first time that this has been shown for lineage III. We thus suggest that the lack of human disease from lineage III LACV is not due to poor transmission ability in local mosquitoes.

## 1. Introduction

La Crosse virus (LACV), first isolated in 1960 from a fatal human case in La Crosse, Wisconsin, has severe clinical manifestations that occur principally in children [1]. LACV is now the leading cause of arboviral pediatric encephalitis in the USA, La Crosse encephalitis, which occurs annually, predominantly in Appalachia and the Midwest [2,3,4]. This is a reportable disease with clinical manifestations including headache, fever, vomiting, seizures, disorientation, swelling of the brain and meninges, among other neurological symptoms that primarily affect children under 16 years of age, of which there are 30–90 cases reported to the CDC annually [5,6,7,8]. LACV can also cause a non-encephalitic clinical infection, likely vastly underreported, characterized by fever and headache, or often entirely asymptomatic [5,6,7,8]. LACV seroprevalence in humans is suspected to be as high as 17.7% [9].

LACV is a tripartite, negative-sense RNA virus in the California serogroup of the *Orthobunyavirus* genus of the *Bunyavirales* order [10,11]. LACV is maintained in a sylvatic cycle predominantly in hardwood forests using *Aedes* spp. mosquito vectors and Sciuridae species, such as chipmunks and squirrels, that serve as reservoir hosts [12,13,14,15,16,17]. *Aedes triseriatus* (Diptera: Culicidae, Say 1823) is the primary vector of LACV in endemic areas, as indicated by the continued isolation of LACV from field-caught mosquitoes, laboratory experiments, and the ecology of the vector [18,19,20]. LACV has been shown to overwinter in *Ae. triseriatus* eggs, where emerging adults in the spring are infected [18,19,21]. LACV in *Ae. triseriatus* mosquitoes can be transmitted via horizontal and vertical transmission [15,22,23,24]. Like other *Orthobunyaviruses*, LACV can be maintained through transovarial transmission (TOT) from mother to offspring, a form of vertical transmission [15,22,23,24,25]. TOT is likely the integral overwintering mechanism for the persistence of LACV in mosquito eggs, a phenomenon that has been experimentally demonstrated [19,26,27]. LACV can also be transmitted sexually from transovarially infected males to females and subsequently into offspring [28,29]. However, this route is not addressed in the current study.

**Figure 1:**
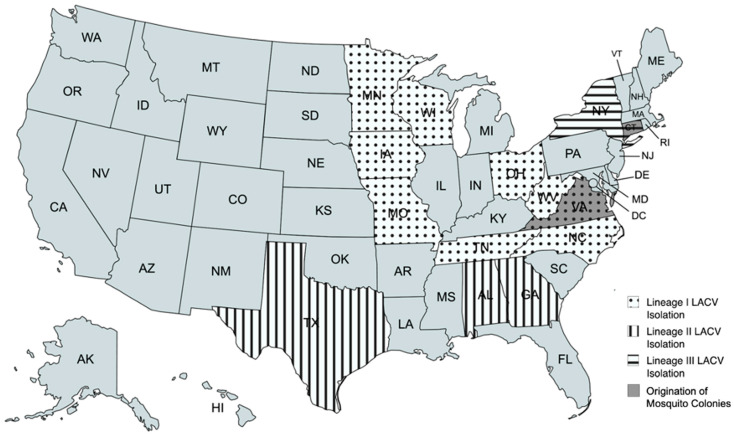
Map of the United States, with patterns indicating states where LACV lineage isolations have been made and gray indicating the states where mosquito colonies were acquired for this study. Created with mapchart.net.

Historically, LACV has encompassed two genetically distinct lineages that are geographically isolated in the midwestern (lineage I), the Appalachian (lineage I), and southern (lineage II) regions of the USA [25,30] (Figure 1). A lineage III strain was first isolated from a pool of *Aedes triseriatus* mosquitoes captured in Connecticut in 2005 [30]; since then, several additional lineage III isolations have been made in the Northeast from a wider range of mosquito species [25,30]. LACV infections are generally associated with the geographic location where the virus was acquired, and most cases of LACV thus far have been associated with LACV lineage I [12].

To date, there have not been any known LACV clinical cases associated with lineage III; even though there have been two cases of LACV disease in the Northeast, both were acquired elsewhere [25], RI Dept. of Health, Pers. Comm. With an apparent entomological risk, as determined by the continued isolation of lineage III viral strains from mosquitoes in the Northeast, the lack of human cases is unusual. The prevailing hypotheses for this discrepancy are (i) underdiagnosis in cases of human disease or lack of detection in humans, (ii) reduced virulence in lineage III LACV, (iii) low prevalence of lineage III in local mosquito and host animal populations, or (iv) reduced vector competence in local mosquito populations [25]. Here, we explore the latter hypothesis and test the vector competence of *Aedes* spp. mosquitoes for lineage III LACV compared to lineage I and II strains, considering two candidate mosquito vector species from two different U.S. states (one population from a historical lineage I range, Virginia, and the other from the lineage III range, Connecticut).

It is suggested that invasive mosquito species, specifically *Aedes albopictus* (Diptera: Culicidae, Skuse 1894), contribute to LACV spread, as they are competent vectors of LACV and are known to feed on humans [25,31,32,33]. LACV has been isolated from wild populations of both *Ae. albopictus* and *Ae. triseriatus* in Tennessee, where the maximum likelihood estimates [number of infected mosquitoes per 1000] for LACV were higher for *Ae. albopictus* than for *Ae. triseriatus* [34]. This highlights the potential role of *Ae. albopictus* in LACV transmission. Here, we focus on the vector competency of *Ae. triseriatus* as a native vector of LACV and *Ae. albopictus* as an invasive vector species.

Evidence shows that the sympatric ‘geographic pairing’ between mosquitoes and viruses from the same region affects vector competency [35]. Susceptibility to LACV infection and horizontal transmission ability have been shown to be higher in mosquitoes from non-endemic LACV areas and are believed to be an evolutionary adaptation towards LACV resistance in mosquitoes from endemic regions [35]. The ability of a virus to be more efficiently transmitted when geographically [allopatric] mismatched highlights the need for further vector competency studies on invasive mosquito species as they are exposed to novel pathogens. However, it was previously demonstrated that ‘geographic pairing’ had an inverse effect on the capacity for LACV vertical transmission, where sympatric pairing increased vertical transmission, indicating that vertical transmission is a more effective transmission mechanism in endemic regions, but horizontal transmission is more effective in non-endemic regions [35,36,37]. In addition to the concern of new vectors migrating to new areas, invasive vectors may be more susceptible to infection or more easily transmit the virus, which has been demonstrated in other pathogen–vector systems. In *Ae. albopictus* infected with dengue virus (DENV), the geographic origin of the mosquitoes has a similar effect on vector competency, where those exposed to DENV more often exhibit lower horizontal vector competency [38]. The ability of a virus to be more efficiently transmitted horizontally when not geographically paired with the vector further highlights the need for vector competency studies on different populations of mosquitoes, including invasive mosquito species exposed to novel pathogens.

Here, we explored the efficacy of mosquito acquisition and transmission of lineage III LACV compared to lineage I and II to determine the role of vector competency in the lack of human cases of lineage III LACV. Vector competency was explored here as the potential for mosquitoes to transmit the virus horizontally by examining the presence of virus in bodies, legs, and saliva of mosquitoes exposed to LACV, and vertically, through the presence of virus in the ovaries of mosquitoes exposed to LACV. We considered the mosquito species, geographic population, and virus lineage as factors of vector competency.

## 2. Materials and Methods

### 2.1. Mosquito Populations

Connecticut *Ae. albopictus* and *Ae. triseriatus* mosquito eggs were acquired from established laboratory colonies at the Connecticut Agricultural Experiment Station (CAES—New Haven, CT, USA). From Virginia, *Ae. albopictus* and *Ae. triseriatus* eggs were field collected using constructed oviposition traps (small black cups or basins lined with seed germination paper and water) in Blacksburg, VA (Figure 1). After at least a 2-week incubation at ambient temperatures, egg papers were hatched in deionized water, and larvae were provided a 3:2 liver powder–Brewer’s yeast mixture as food. Mosquitoes were reared to adulthood, with all life stages maintained at 26 °C (22 °C during the scotophase) with a photoperiod of 15 h light (L):9 h dark (D) and 80% relative humidity (RH). All adult mosquitoes were offered 10% sucrose solution ad lib, and populations were maintained by artificial blood-feeding using a Hemotek feeding system (Hemotek, Blackburn, UK) with defibrinated bovine or sheep blood (Lampire Biological Laboratories, Pipersville, PA, USA, or Hemostat Laboratories, Dixon, CA, USA) through a hog casing membrane. Due to challenges in producing viable offspring, *Ae. triseriatus* (from both geographical regions) were force-mated by attaching decapitated males to small pins and mating them to females anesthetized on ice, *Ae. albopictus* colonies were free-mating and did not require force-mating. The exact generation of both colonies is unknown, but is estimated to be approximately generation 10.

As a risk group 2 pathogen, LACV-work took place in an ACL-3 insectary similarly maintained at 26 °C using a photoperiod of 15:9 L:D at 80% RH.

Oral Infection: Prior to viral exposure, adult female mosquitoes were allowed 96 h for maturation and mating after eclosion from pupae and sugar-starved for 24 h. Mosquitoes were presented with an infectious blood meal using a Hemotek feeding system as above. Approximately 5 × 10^5^ PFU/mL of a relevant strain of virus, plus 1% 0.2 mM ATP (to increase gustation), were included in each bloodmeal. Four LACV virus strains—NC97 (lineage I), TX09 (II), NY05 (III), and CT18 (III)—representing all three lineages were used. An aliquot was taken from each bloodmeal to confirm the initial viral titer, using plaque assay methods referenced in Payne et al. (2006) [39]. Allowing one hour for feeding, or until most mosquitoes were fully engorged, the engorged mosquitoes were separated from non-engorged individuals.

Intrathoracic Inoculation: Before inoculation, mosquitoes were similarly allowed to mature for at least 96 h post-emergence. Mosquitoes were inoculated intrathoracically with approximately 20 pfu (200 nL of approximately 1 × 10^5^ pfu/mL) of the virus using a Nanoject III microinjector (Drummond, Broomall, PA, USA). Again, each of the four LACV virus strains—NC97 (lineage I), TX09 (II), NY05 (III), and LV1864 (III)—was used to represent all three lineages.

### 2.2. Viral Transmission Assays

Engorged or inoculated mosquitoes were housed in small (1-gallon) plastic buckets with a mesh screen lid and provided 10% sucrose ad lib. After a 14-day extrinsic incubation period (EIP), the bodies, legs, and saliva of each mosquito were harvested and assessed for virus presence as indicators of infection, dissemination, and ability to transmit, respectively. Ovaries were harvested as a proxy for the capacity for TOT due to challenges in rearing offspring from infected mothers. Briefly, mosquitoes were immobilized on ice; then, salivary secretions were acquired via forced salivation by inserting the proboscis into a 10 μL micropipette tip capillary containing 5 μL of a 1:1 ratio of 50% sucrose and fetal bovine serum (FBS), with a small drop of food coloring for visualization. After 30 min, the contents of the micropipette tip were transferred into 200 μL of Mosquito Diluent (Dulbecco’s phosphate-buffered saline (dPBS) containing 20% FBS, 100 units/mL penicillin, 100 μg/mL streptomycin, and 2.5 μg/mL amphotericin B). Legs were removed from each mosquito body, and ovaries were dissected. Bodies, legs, and ovaries were placed in individual tubes containing a glass bead and 300μL of mosquito diluent as described above. Samples of mosquito bodies, legs, and ovaries were homogenized using a TissueLyser II homogenizer (Qiagen, Hilden, Germany) at 24 Hz for 30 s twice before centrifugation at 8000 rpm and 4 °C for 4 min.

Mosquito body, leg, and ovary samples were each inoculated onto a confluent monolayer of Vero-76 cells (ATCC, Manassas, VA, USA), incubated at 37 °C with a 5% CO_2_ environment for 1 h. An overlay of 1:5 2% Seakem Agarose (Lonza, Basal, Switzerland): Dulbecco’s Modified Eagle’s Medium (DMEM) (supplemented with 2% FBS, 100 units/mL penicillin, 100 μg/mL streptomycin, and 2.5 μg/mL amphotericin B or 500 μg/mL gentamicin, the latter introduced to address contamination), hereafter referred to as reduced-DMEM, was applied to the sample plates. Plates were incubated at 37 °C and 5% CO_2_ for three days for plaque development, then fixed using 10% formaldehyde solution, and stained with 0.1% crystal violet diluted in 10% phosphate-buffered formalin. Saliva samples were inoculated onto confluent Vero-76 cells as above, but a liquid overlay consisting of reduced-DMEM was used instead, and cells were monitored for cytopathic effect (CPE) for ten days.

### 2.3. Statistical Analyses

Population rates were calculated as the number of positive bodies, legs, ovaries, and salivary secretions of the total number of mosquitoes tested. Because dissemination to the legs is dependent upon midgut infection, and dissemination to secondary tissues (such as the salivary glands and ovaries) is dependent upon this initial dissemination, modified rates were also included. Modified rates were calculated as the number of positive legs that resulted from mosquitoes with midgut infections, and the number of positive salivary secretions or ovaries from those with disseminated infections. Differences in infection, dissemination, ability to transmit, potential for vertical transmission, and ovary titers between LACV groups and mosquito species were analyzed using two-way ANOVA followed by Tukey’s multiple comparisons in GraphPad Prism (version 10.1.1 for Mac; GraphPad Software, Boston, MA, USA). Geographic and species analyses were conducted using two-way ANOVA followed by Tukey’s multiple comparisons in GraphPad Prism. Transmission barriers were identified as modified rates less than 0.75 (25% reduction).

## 3. Results

### 3.1. LACV Competency in Connecticut Aedes triseriatus

#### 3.1.1. Oral Infection

Data could not be obtained for this population of the species due to a lack of survival in mosquitoes.

#### 3.1.2. Intrathoracic Inoculation

**Figure 2:**
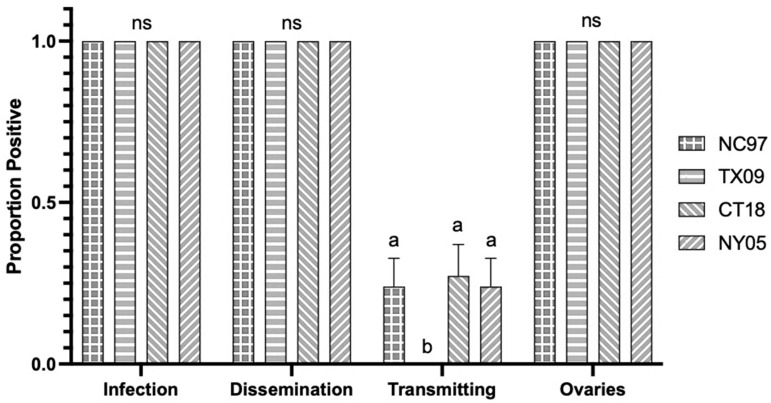
Connecticut *Aedes triseriatus* LACV infection, dissemination, transmission ability, and ovary infection following intrathoracic inoculation of virus expressed as the proportion positive. Letters indicate the significance (*p* < 0.05) between virus strains within each transmission parameter (ANOVA, Tukey’s multiple comparisons); “ns” indicates non-significance.

**Figure 3:**
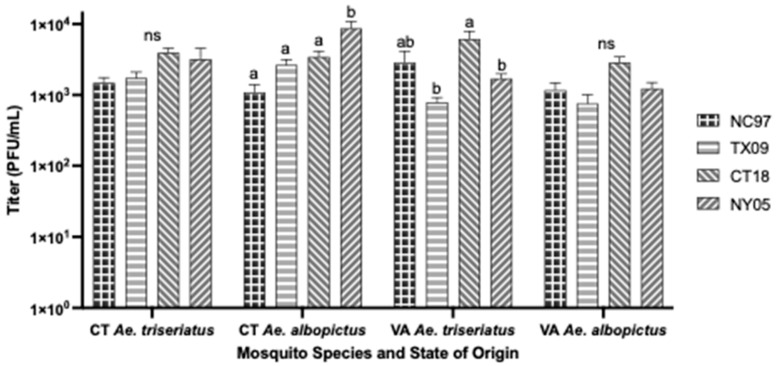
LACV-infected ovary titers for all species from both states of origin following intrathoracic inoculation, expressed as the average titer of positive ovaries. Error bars indicate standard error. Letters indicate the significance (*p* < 0.05) between virus strains within each mosquito species (ANOVA, Tukey’s multiple comparisons); “ns” indicates non-significance.

There were no significant differences in infection rates, dissemination rates, or rates of viral-positive ovaries [proxy for TOT] between strains of LACV (Table A1, with rates shown graphically in Figure 2. Saliva infection rate [horizontal transmission] for lineage II strain TX09 was significantly lower than the rates seen for strains NC97, CT18, and NY05 (Figure 2 and Table A1) (Tukey’s multiple comparisons; NC97 *p*-value < 0.0001, q = 7.241, 95%; CT18 *p*-value < 0.0001, q = 7.917; NY05 *p*-value < 0.0001, q = 7.241). There were no significant differences in ovary titers based on virus strain (Figure 3). Although CT *Ae. triseriatus* showed salivary gland barriers to infection with all lineages; lineage III was transmitted (salivary infection rates) at rates similar to lineage I and II. Other barriers could not be determined because of the absence of oral infection data for this species only.

### 3.2. LACV Competency in Connecticut Aedes albopictus

#### 3.2.1. Oral Infection

**Figure 4:**
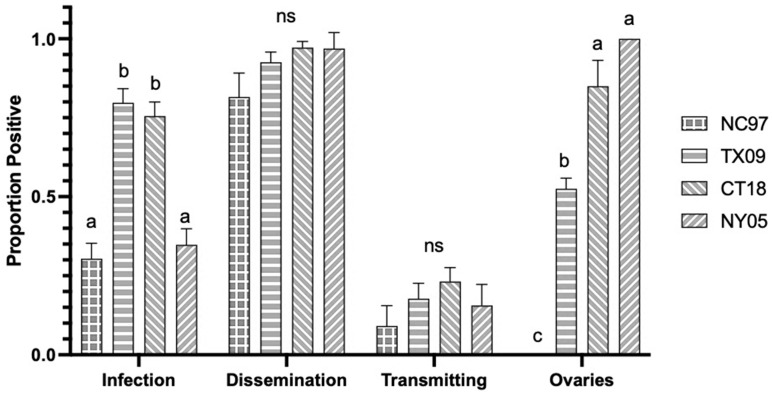
Connecticut *Aedes albopictus* LACV infection, dissemination, transmission ability, and ovary infection following oral feeding of virus expressed as the proportion positive. Letters indicate the significance (*p* < 0.05) between virus strains within each transmission parameter (ANOVA, Tukey’s multiple comparisons); “ns” indicates non-significance.

**Figure 5:**
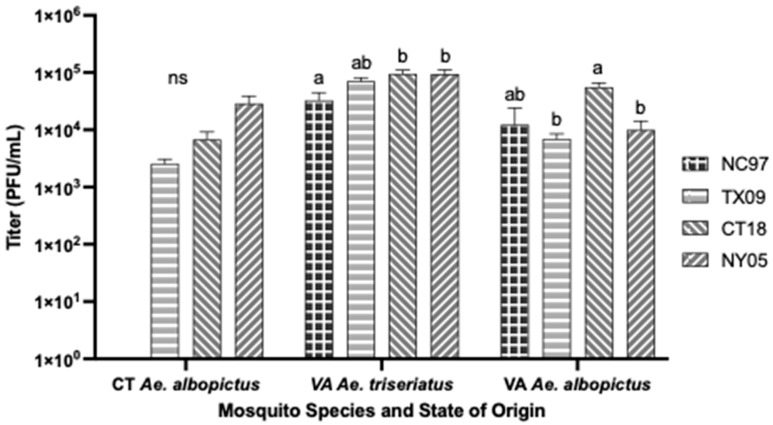
LACV-infected ovary titers for all species from both states of origin following oral infection, expressed as the average titer of positive ovaries. Error bars indicate standard error. Letters indicate the significance (*p* < 0.05) between virus strains within each mosquito species (ANOVA, Tukey’s multiple comparisons); “ns” indicates non-significance.

Rates are listed in Table A2, with modified rates shown graphically in Figure 4. Lineage II TX09 and lineage III CT18 showed significantly higher rates of infection than lineage I NC97 and lineage III NY05 (Tukey’s multiple comparisons; TX09:NC97 *p*-value < 0.0001, q = 12.32; TX09:NY05 *p*-value < 0.0001, q = 11.30; CT18:NC97 *p*-value < 0.0001, q = 11.59; CT18:NY05 *p*-value < 0.0001, q = 10.53). There were no significant differences in dissemination rates or rates of positive salivary secretions between strains. Lineage I strain NC97 had significantly lower rates of infected ovaries (no positive ovaries from this group) than all other groups (Tukey’s multiple comparisons; NC97:TX09 *p*-value < 0.0001, q = 7.499; NC97:CT18 *p*-value < 0.0001, q = 10.43; NC97:NY05 *p*-value < 0.0001, q = 7.653), while CT18 and NY05 had significantly higher rates of infected ovaries than TX09 (Tukey’s multiple comparisons; CT18:TX09 *p*-value = 0.0083, q = 4.499; NY05:TX09 *p*-value = 0.0371, q = 7.499) (Figure 4 and Table A2). There were no significant differences between ovary titers based on virus strain (Figure 5).

#### 3.2.2. Intrathoracic Inoculation

**Figure 6:**
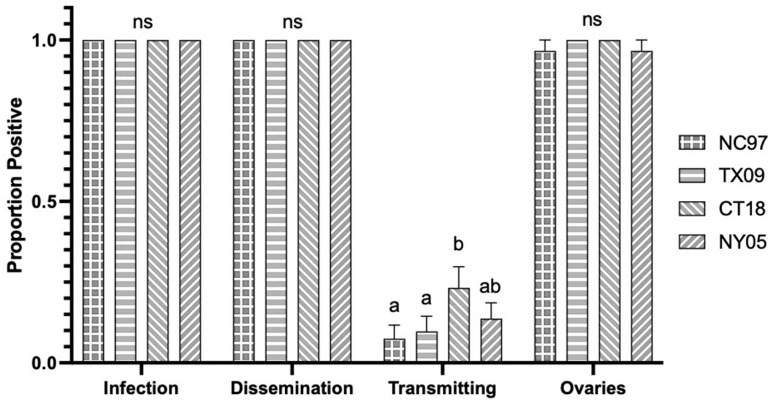
Connecticut *Aedes albopictus* LACV infection, dissemination, transmission ability, and ovary infection following intrathoracic inoculation of virus expressed as the proportion positive. Letters indicate the significance (*p* < 0.05) between virus strains within each transmission parameter (ANOVA, Tukey’s multiple comparisons); “ns” indicates non-significance.

Rates are listed in Table A3, with rates shown graphically in Figure 6. There were no significant differences in infection rates, dissemination rates, or rates of positive ovaries between strains of LACV inoculated into CT *Ae. albopictus*. Lineage III strain CT18 had a significantly higher salivary infection rate than lineage I NC97 and lineage II TX09 (Tukey’s multiple comparisons; CT18:NC97 *p*-value = 0.0009, q = 5.385; CT18:TX09 *p*-value = 0.0060, q = 4.644) (Figure 6 and Table A3). Lineage III strain NY05 produced significantly higher ovary titers compared to the other strains (Tukey’s multiple comparisons; NY05:NC97 *p*-value < 0.0001, q = 6.255; NY05:TX09 *p*-value = 0.0034, q = 4.885; NY05:CT18 *p*-value = 0.0218, q = 4.067) (Figure 3).

### 3.3. Variation Between Mosquito Species from Connecticut

Contrasting the above results for the two different mosquito species (*Ae. triseriatus* and *Ae. albopictus*) originating in Connecticut, there were no significant differences in infection rates, dissemination rates, or rates of positive ovaries between species (this is based on intrathoracic inoculation testing). NC97 had significantly higher transmission rates of infected salivary secretions in *Ae. triseriatus* mosquitoes than *Ae. albopictus* (Tukey’s multiple comparisons; *p*-value = 0.0115, q = 4.946). NY05 in *Ae. albopictus* had significantly higher ovary titers than *Ae. triseriatus* mosquitoes (Tukey’s multiple comparisons; *p*-value = 0.0129, q = 4.313).

### 3.4. LACV Competency in Virginia Aedes triseriatus

#### 3.4.1. Oral Infection

**Figure 7:**
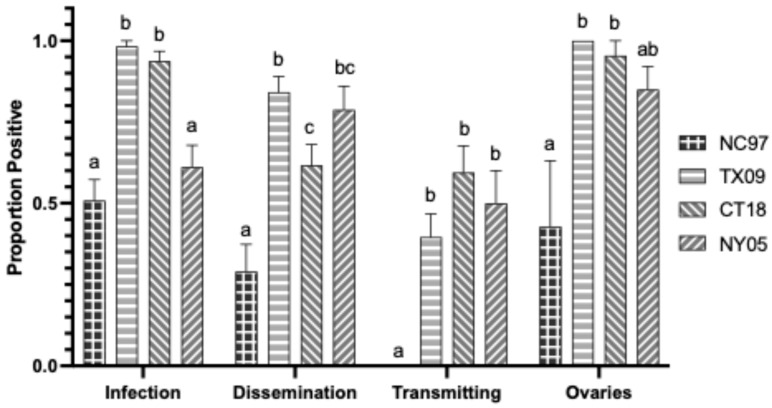
Virginia *Aedes triseriatus* LACV infection, dissemination, transmission ability, and ovary infection following oral feeding of virus expressed as the proportion positive. Letters indicate the significance (*p* < 0.05) between virus strains within each transmission parameter (ANOVA, Tukey’s multiple comparisons).

Rates are listed in Table A4, with modified rates shown graphically in Figure 7. NC97 and NY05 strains had significantly lower infection rates than TX09 and CT18 (Tukey’s multiple comparisons; NC97:TX09 *p*-value < 0.0001, q = 9.031; NC97:CT18 *p*-value < 0.0001, q = 8.373; NY05:TX09 *p*-value < 0.0001, q = 6.859; NY05:CT18 *p*-value < 0.0001, q = 6.165). NC97 has significantly lower rates of dissemination than all other strains (Tukey’s multiple comparisons; NC97:TX09 *p*-value < 0.0001, q = 8.630; NC97:CT18 *p*-value = 0.0017, q = 5.149; NC97:NY05 *p*-value < 0.0001, q = 6.943). TX09 had significantly higher rates of dissemination than CT18 (Tukey’s multiple comparisons; *p*-value = 0.0145, q = 4.254). NC97 has reduced horizontal transmission rates of infected saliva compared to all other groups (Tukey’s multiple comparisons; NC97:TX09 *p*-value = 0.0369, q = 3.803; NC97:CT18 *p*-value = 0.0005, q = 5.583; NC97:NY05 *p*-value = 0.0081, q = 4.512). NC97 also had a reduced ovarian infection rate compared to TX09 and CT18 (Tukey’s multiple comparisons: NC97:TX09 *p*-value = 0.0052, q = 4.702; NC97:CT18 *p*-value = 0.0153, q = 4.230), and NC97 further had reduced ovarian titers compared to lineage III strains CT18 and NY05 (Tukey’s multiple comparisons: NC97:CT18 *p*-value = 0.0046, q = 4.794; NC97:NY05 *p*-value = 0.0064, q = 4.653) (Figure 7).

#### 3.4.2. Intrathoracic Inoculation

**Figure 8:**
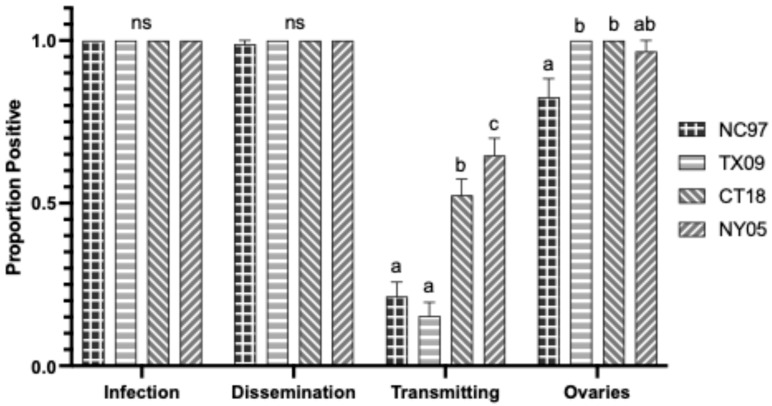
Virginia *Aedes triseriatus* LACV infection, dissemination, transmission ability, and ovary infection following intrathoracic inoculation of virus expressed as the proportion positive. Letters indicate the significance (*p* < 0.05) between virus strains within each transmission parameter (ANOVA, Tukey’s multiple comparisons); “ns” indicates non-significance.

Rates are listed in Table A5 and shown graphically in Figure 8. There were no significant differences in infection and dissemination rates between LACV strains. Strains NC97 and TX09 had significantly lower transmission rates of positive saliva than lineage III strains CT18 and NY05 (Tukey’s multiple comparisons; NC97:CT18 *p*-value < 0.0001, q = 11.94; NC97:NY05 *p*-value < 0.0001, q = 15.73; TX09:CT18 *p*-value < 0.0001, q = 15.73; TX09:NY05 *p*-value < 0.0001, q = 17.32). TX09 and CT18 had significantly higher TOT rates of positive ovaries than NC97 (Tukey’s multiple comparisons; TX09:NC97 *p*-value = 0.0190, q = 4.122; CT18:NC97 *p*-value = 0.0028, q = 4.936) (Figure 8 and Table A5). CT18 had significantly higher ovarian titers than TX09 and NY05 (Tukey’s multiple comparisons; CT18:TX09 *p*-value = 0.0018, q = 5.130; CT18:NY05 *p*-value = 0.0144, q = 4.262) (Figure 3).

### 3.5. LACV Competency in Virginia Aedes albopictus

#### 3.5.1. Oral Infection

**Figure 9:**
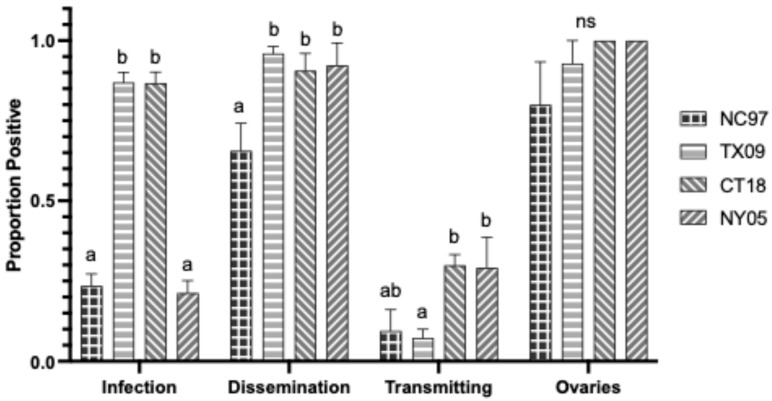
Virginia *Aedes albopictus* LACV infection, dissemination, transmission ability, and ovary infection following oral feeding of virus expressed as the proportion positive. Letters indicate the significance (*p* < 0.05) between virus strains within each transmission parameter (ANOVA, Tukey’s multiple comparisons); “ns” indicates non-significance.

Rates are listed in Table A6, with modified rates shown graphically in Figure 9. TX09 and CT18 had significantly higher rates of infection than NC97 and NY05 (Tukey’s multiple comparisons; TX09:NC97 *p*-value, 0.0001, q = 19.43; TX09:NY05 *p*-value, 0.0001, q = 19.60; CT18:NC97 *p*-value, 0.0001, q = 18.51; CT18:NY05 *p*-value, 0.0001, q = 18.72). NC97 had significantly lower rates of dissemination than all other strains (Tukey’s multiple comparisons; NC97:TX09 *p*-value = 0.0003, q = 5.804; NC97:CT18 *p*-value = 0.0055, q = 4.671; NC97:NY05 *p*-value = 0.0289, q = 3.922). TX09 had significantly lower horizontal transmission rates of positive saliva secretions than lineage III strains CT18 and NY05 (Tukey’s multiple comparisons; TX09:CT18 *p*-value = 0.0003, q = 5.727; TX09:NY05 *p*-value = 0.0430, q = 3.720). There were no significant differences in the rate of positive ovaries for any strain (Figure 9 and Table A6). CT18 had significantly higher ovarian titers than TX09 and NY05 (Tukey’s multiple comparisons; CT18:TX09 *p*-value = 0.0090, q = 4.505; CT18:NY05 *p*-value = 0.0301, q = 3.930) (Figure 5).

#### 3.5.2. Intrathoracic Inoculation

**Figure 10:**
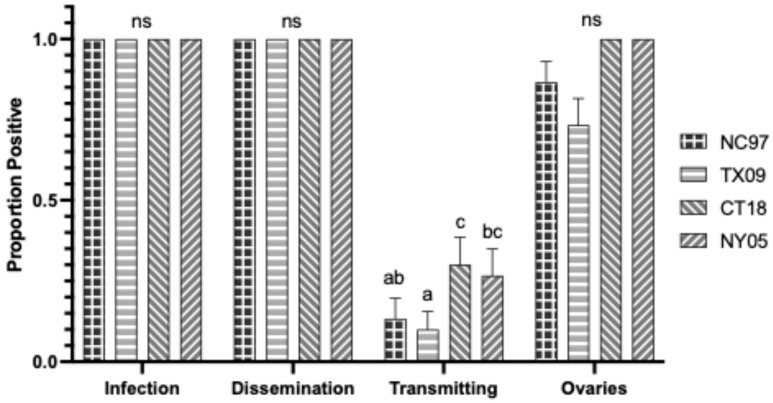
Virginia *Aedes albopictus* LACV infection, dissemination, transmission ability, and ovary infection following intrathoracic inoculation of virus expressed as the proportion positive. Letters indicate the significance (*p* < 0.05) between virus strains within each transmission parameter (ANOVA, Tukey’s multiple comparisons); “ns” indicates non-significance.

Rates are listed in Table A7 and shown graphically in Figure 10. There were no significant differences in infection rates, dissemination rates, or ovary infection rates between LACV strains. Transmission-wise, the lineage III strain CT18 had significantly higher rates of virus-positive saliva than NC97 and TX09 (Tukey’s multiple comparisons; CT18:NC97 *p*-value = 0.0421, q = 3.738; CT18:TX09 *p*-value = 0.0087, q = 4.486); NY05 had higher transmission rates of positive saliva than TX09 (Tukey’s multiple comparisons; NY05:TX09 *p*-value = 0.0421, q = 3.738) (Figure 10 and Table A7). There were no significant differences in the ovary titers based on virus strain (Figure 3).

### 3.6. Variation Between Mosquito Species from Virginia

We contrasted the above results for the two different mosquito species (*Ae. triseriatus* and *Ae. albopictus*) originating in Virginia.

#### 3.6.1. Oral Infection

*Ae. triseriatus* infected with NC97 and NY05 had significantly higher infection rates than *Ae. albopictus* (Tukey’s multiple comparisons; NC97 *p*-value < 0.0001, q = 6.582; NY05 *p*-value < 0.0001, q = 9.050). *Ae. albopictus* infected with NC97 and CT18 had significantly higher dissemination rates than *Ae. triseriatus* (Tukey’s multiple comparisons; NC97 *p*-value = 0.0035, q = 5.397; CT18 *p*-value = 0.0002, q = 6.375). *Ae. triseriatus* infected with TX09 and CT18 had significantly higher horizontal transmission rates of infected salivary secretions than *Ae. albopictus* (Tukey’s multiple comparisons; TX09 *p*-value < 0.0001, q = 6.789; CT18 *p*-value = 0.0027, q = 5.498). While there were no significant differences in vertical transmission ovarian infection rates between the two vector species, TX09, CT18, and NY05 had significantly higher ovarian titers in *Ae. triseriatus* than in *Ae. albopictus* (Tukey’s multiple comparisons; TX09 *p* < 0.0001, q = 6.377; CT18 *p* = 0.0098, q = 4.178; NY05 *p* < 0.0001, q = 7.446).

#### 3.6.2. Intrathoracic Inoculation Variation

There were no significant differences in infection or dissemination rates between inoculated species from Virginia for any LACV virus strain. CT18 and NY05 showed higher rates of infected salivary secretions in *Ae. triseriatus* than *Ae. albopictus* (Tukey’s multiple comparisons; CT18 *p*-value = 0.0004, q = 6.118; NY05: *p*-value < 0.0001, q = 10.07). TX09 had higher rates of ovarian infection in *Ae. triseriatus* than in *Ae. albopictus* mosquitoes (Tukey’s multiple comparisons; *p* = 0.0037, q = 5.376). There were no differences in ovary titers between VA mosquito species.

### 3.7. Overall Geographic Variation in LACV Vector Competency

#### 3.7.1. *Aedes triseriatus* Intrathoracic Inoculation

There were no significant differences in infection rates, dissemination rates, or rates of positive ovaries between Connecticut- and Virginia-inoculated *Ae. triseriatus* mosquitoes. However, the vertical transmission rate of positive salivary secretions was higher in VA mosquitoes inoculated with TX09, CT18, and NY05 than in CT mosquitoes (Tukey’s multiple comparisons; TX09 *p*-value = 0.029, q = 4.547; CT18 *p* = 0.0002, q = 6.378; NY05 *p* < 0.0001, q = 10.59). There were no significant differences in ovary titers based on state of origin.

#### 3.7.2. *Aedes albopictus* Intrathoracic Inoculation

There were no significant differences in infection rates, dissemination rates, or rates of positive salivary secretions between Connecticut- and Virginia-inoculated *Ae. albopictus* mosquitoes. Considering the proxy for vertical transmission, TX09 had a significantly higher rate of infected ovaries in CT *Ae. albopictus* than in VA *Ae. albopictus* (Tukey’s multiple comparisons, *p* = 0.0003, q = 6.285). Lineage III NY05 had significantly higher ovary titers in CT *Ae. albopictus* than in VA *Ae. albopictus* (Tukey’s multiple comparisons, *p* = 0.0001, q = 6.138) (Figure 3).

#### 3.7.3. *Aedes albopictus* Oral Infection

There were no significant differences in infection rates, dissemination rates, and rates of positive salivary secretions between Connecticut- and Virginia- *Ae. albopictus* exposed to LACV via oral infection. However, Virginia mosquitoes bloodfed with NC97 and TX09 produced significantly higher rates of ovary infection than Connecticut *Ae. albopictus* (Tukey’s multiple comparisons; NC97 *p*-value < 0.0001, q = 8.053; TX09 *p* = 0.0102, q = 4.990). Virginia *Ae. albopictus* bloodfed with CT18 had higher ovary titers than Connecticut mosquitoes (Tukey’s multiple comparisons, *p* = 0.0016, q = 4.960) (Figure 5).

## 4. Discussion

Addressing the potential public health risk posed by novel lineage III LACV viral strains identified in the northeast USA, we examined the role of the mosquito as a vector for these viral strains. LACV lineage III has been isolated from mosquitoes, but there has yet to be a clinical infection associated with this third lineage [30]. Here, we sought to investigate vector competency for different lineages of LACV, to consider the hypothesis that a lack of human incidence in the Northeast USA could be due to mosquito species having a reduced capacity to transmit strains of lineage III. We demonstrated that, although there was variation between strains, lineage III was as competent for the potential for horizontal transmission (saliva) and vertical transmission (ovaries) as lineage I and II. This suggests that a lack of vector competency does not explain the absence of clinical cases of lineage III. We also highlight the presence of another tissue barrier to infection, an ovarian infection barrier.

There are three main steps to arboviral transmission in the mosquito vector: (1) midgut infection, (2) dissemination from the midgut to secondary tissues, and (3) invasion into the salivary glands [40,41]. Mosquitoes contain barriers to arboviral infection that correlate with these steps to arboviral transmission, and viruses can evade these barriers in their corresponding vectors [40]. In the midgut, arboviruses must infect the mosquito midgut (midgut infection barrier, MIB) and escape the midgut to disseminate to other tissues (midgut escape barrier, MEB) [40]. The MIB can be caused by either the virus’s inability to infect the epithelial cells or its failure to replicate in these cells [40]. Both midgut barriers are dose-dependent [40]. In *Ae. triseriatus*, larger mosquitoes have a stronger MIB to LACV infection than smaller females [40,42]. Our rearing methods ensured that mosquitoes were consistent in size. *Aedes triseriatus* also expresses a significant midgut escape barrier (MEB) in which dissemination rates of the virus are reduced when mosquitoes are fed an infectious blood meal [43]. Although this was not previously explored in *Ae. albopictus*, the current study also shows prevalent MEBs in both species, as detailed below [40,44].

Our data indicate that the ability of a virus to overcome mosquito infection barriers varies according to mosquito species, state of origin, and virus strain. Barriers were identified by proportions of 0.75 or less for each transmission parameter. Due to the lack of an oral feeding comparison for CT *Ae. triseriatus*, it is unclear whether midgut barriers exist. In the other species and geographic origins, the presence of midgut barriers (MIB and MEB) varied between groups. However, NC97 and NY05 consistently encountered MIBs in all groups, which may indicate that midgut infection barriers partially modulate transmission in these two strains.

In CT *Ae. albopictus*, NC97, and TX09 demonstrated a reduced ability to infect the ovaries, yet the virus disseminated through the hemolymph at high rates (shown by infected legs), indicating that there is no MEB. The absence of infected ovaries despite widespread systemic infection suggests that a barrier exists within the ovaries that limits vertical transmission. Although several potential barriers to ovarian infection have been proposed, the exact mechanisms remain unclear and are not well understood [44].

As with the midgut, mosquitoes have barriers in the salivary glands that affect virus transmission. The salivary gland infection barrier (SGIB) prevents the glands from becoming infected, while the salivary gland escape barrier (SGEB) stops viruses from being released in saliva during feeding [39]. Both types of barriers have been shown for LACV in the *Triseriatus* group mosquitoes, *Ae. brelandi and Ae. hendersoni* [42]. In our study, all species and virus strains showed SGBs after blood feeding, and this was confirmed by inoculation methods that bypassed midgut barriers. The consistent presence of SGBs suggests they play an important role in blocking transmission. However, we did not determine whether the barrier was due to infection or escape, so further research is needed. A persistent salivary gland barrier (SGB) significantly reduced horizontal transmission across all mosquito species, virus strains, and geographic origins, and its effect was greater than that of the apparent ovarian infection barrier. Previous studies, in addition to our findings, show that virus-positive salivary secretions occur at lower rates than ovarian infections [45]. Together, the presence of an SGB and high ovarian infection rates indicate that vertical transmission is more efficient than horizontal transmission in the species and strains examined. Earlier work demonstrated that LACV can persist for up to four years through transovarial transmission (TOT) alone in *Ae. triseriatus*, underscoring the importance of vertical transmission in LACV maintenance [22]. Infection, dissemination, transmission, and vertical transmission rates following oral infection with LACV vary in previous studies utilizing lineage I LACV strains. Infection rates reported for *Ae. triseriatus* range between 57% and 94%; our rates of 51% to 98% for lineage III LACV are consistent with these rates [16,45,46]. Previously reported dissemination rates for *Ae. triseriatus* range from 45% to 86%, while ours are lower at 29% to 84% (14% to 83% of the population) [16,43,45,46]. Previously reported vertical transmission rates were approximately 56% for *Ae. triseriatus*, while ours ranged from 0% to 59% (0% to 34% of the population) [43]. Note that other studies of competency have primarily utilized lineage I strains of LACV (Bara et al., 2016 [46] used lineage II), which may vary from other lineage strains and highlight the need to determine accurate transmission rates specifically for lineage III in the face of a potential human pathogen.

In *Ae. albopictus*, infection rates following oral infection reported elsewhere have ranged between 42% and 77% compared to our rates of 21% to 87% [16,45,46]. Reported rates of dissemination range from 18% to 70%, while ours ranged from 65% to 97% (15% to 83% of the population). There were no previous rates of LACV-positive salivary secretions for *Ae. albopictus* and our data ranged from 7% to 30% (1% to 23% of the population). However, when comparing rates across studies, it is essential to note that variation in extrinsic incubation periods, which ranged in previous studies from 7 to 14 days in previous studies, may affect vector competency, resulting in disparate rates.

Few studies compare the rates of LACV infection in inoculated mosquitoes. One significant advantage of inoculation over oral feeding is bypassing the midgut infection and escape barriers, which results in 100% infection and dissemination. Our data were no exception; infection rates were 100% for both populations of *Ae. triseriatus* and *Ae. albopictus* and dissemination rates ranged from 98% to 100% for *Ae. triseriatus* and 100% for *Ae. albopictus*. A 100% dissemination rate for inoculated *Ae. triseriatus* was previously demonstrated [43]. Previously reported rates of virus-positive saliva in *Ae. triseriatus* were 47–79% and in the current study, our transmission rates ranged from 0% to 65% [43,47].

Initial attempts to rear offspring from LACV-infected female mosquitoes were unsuccessful due to low egg viability and larval survival. Therefore, ovarian infection was used as an indicator of the potential to produce virus-positive offspring. Previously, in orally infected *Ae. albopictus*, 100% of ovaries from midgut-infected mosquitoes were infected, and titers ranged from 1 × 10^2^ pfu/mL to 1 × 10^4^ pfu/mL by seven days post-infection [48]. In the current study, orally-infected mosquitoes had ovarian infections ranging from 0% to 100%, with titers ranging from 2.5 × 10^3^ pfu/mL to 2.9 × 10^4^ pfu/mL in CT *Ae. albopictus* mosquitoes and 80% to 100% with titers ranging from 6.9 × 10^3^ pfu/mL to 5.5 × 10^4^ pfu/mL (excluding the zero/lack of titer for NC97) in VA *Ae. albopictus* mosquitoes. Our reported ovarian titers are marginally larger than those previously reported, but this may be explained by our longer extrinsic incubation period of 14 days. In *Ae. triseriatus* mosquitoes from VA, ovarian infection rates ranged between 43% and 100%, with titers ranging from 3.3 × 10^4^ pfu/mL to 9.6 × 10^4^ pfu/mL.

LACV lineage III has also recently been isolated from *Aedes canadensis*, *Aedes cinereus*, and *Aedes trivittatus* in Connecticut [25]; the latter two species have not been previously associated with LACV, but virus isolation alone does not indicate that these species are competent vectors of LACV lineage III. LACV lineage I has been isolated from field-collected *Ae. albopictus* and shown in laboratory studies to be a competent vector for both lineages I and II [32,46,47,49]. In contrast, lineage III has never been isolated from *Ae. albopictus*, and its vector competence for this lineage was previously unknown. Our study demonstrates that *Ae. albopictus* can serve as a potential vector for both horizontal and vertical transmission of LACV lineage III, with no apparent differences in transmission efficiency among lineages or strains.

Previous studies concluded that sympatric matching of mosquito species and virus lineage relevant to the same geographic range resulted in reduced horizontal transmission [35]. However, considering vertical transmission, the ‘geographic pairing’ of viruses and *Ae. triseriatus* mosquitoes from endemic areas can increase capacity [36,37]. Together, this may indicate that LACV vertical transmission is a more effective persistence mechanism in endemic ranges, but horizontal transmission is utilized when mosquito species and viruses are allopatric [35,36,37]. Although there was geographic variation in some variables of vector competence, the current study was unable to demonstrate consistent geographic pairing for horizontal or vertical transmission.

There was no clear pattern in vector competence when comparing *Ae. triseriatus*, the native vector, and *Ae. albopictus*. Studies elsewhere have emphasized the concern regarding invasive non-native vectors and the potential for increased transmission of arboviruses as these species migrate into new areas [16,46]. Prior reports are inconclusive, with some studies demonstrating *Ae. triseriatus* as a better vector of LACV than *Ae. albopictus* [16,45] and others indicating the opposite [46]. The present study found variation between species; however, this variation was largely inconsistent across strains, and thus, we could not conclude one species was a more competent vector for LACV than the other. In the absence of clear differences between groups, it is difficult to make conclusive statements regarding species variation in vector competency.

Our testing was limited to only one strain of lineage I, one strain of lineage II, and two strains of lineage III. With few strains from each lineage used, it is difficult to conclude lineage-specific vector competency. This study was also limited by challenges in maintaining CT *Ae. triseriatus* mosquitoes for further experimentation. The current study employed a temperature of 26 °C with a photoperiod of 15 h of light (L) and 9 h of darkness (D) for all experiments. Previous studies have concluded that vector competency can be affected by temperature [49,50]. Photoperiod also has a profound effect on mosquito biology. *Aedes* spp. mosquitoes in cooler climates, such as *Ae. triseriatus* and *Ae. albopictus*, enter a photoperiod-induced diapause to overwinter as eggs [50]. Based on lineage I, LACV persists through the overwintering of LACV-infected (vertically infected) eggs, and newly emerged adults can infect naïve host species in the spring, thereby maintaining the LACV transmission cycle [19,21,51]. It is thus likely that increases in temperatures and changes in critical photoperiods may alter vector competency for LACV transmission. The use of ovaries as a proxy for vertical transmission also presents a limitation to this study. Although ovaries provide a good approximation for the potential to produce infected offspring, it is impossible to know the viability of those offspring or the rates of infection in them. Our study utilized a single time point of 14 days post-inoculation, and it would be beneficial to explore the influence of the extrinsic incubation period on vector competency to strengthen findings. Although forced salivation is an accepted proxy for the ability to transmit via the bite of an infected mosquito, it was recently demonstrated that forced salivation may not be a reliable measure of this ability [47]. In the present study, dissemination rates, both dissemination to legs and ovaries, were consistently higher for all groups than the infected saliva rates. The use of dissemination rates to approximate the ability to transmit would substantially increase the potential transmission ability in all groups and suggest a more significant human disease risk.

## 5. Conclusions

Overall, we indicate that LACV lineage III can be transmitted horizontally and vertically in all mosquito populations tested here, highlighting its role as a potential human pathogen risk. This is a novel finding revealing the potential vector competency of two prominent mosquito species, *Aedes triseriatus* and *Aedes albopictus*, for which lineage III virus interactions had not been previously investigated. Our data also suggest that transmission of LACV lineage III is not modulated by poor vector competency, rejecting the key hypothesis that mosquitoes are unable to transmit lineage III horizontally, and implying that another hypothesis is responsible for the apparent absence of human cases. Alternative suggestions remaining to be explored include (1) underdiagnosis in cases of human disease or lack of detection in humans, (2) reduced virulence in lineage III LACV (recent research provides evidence for this hypothesis [52]), or (3) low prevalence of lineage III in local mosquito and host animal populations. We recommend further investigation into the biology and ecoepidemiology of lineage III, with future studies to determine the human disease risk associated with LACV lineage III infection.

## Data Availability

The raw data supporting the conclusions of this article will be made available by the authors upon request.

## Appendix A

**Table A1:** Connecticut *Aedes triseriatus* LACV-inoculation data by trial expressed as proportion infected out of total number tested. N values in parentheses. Values in parentheses in ovary titer rows indicate the standard error. Letters indicate significance between strains within each transmission parameter in each trial (ANOVA, Tukey’s multiple comparisons).

CT *Aedes triseriatus* LACV Exposure by Intrathoracic Inoculation					
		Virus Strain (Lineage)			
Trial	Transmission Parameter	NC97 (I)	TX09 (II)	CT18 (III)	NY05 (III)
1	Infection (bodies)	1.00 (25/25) a	1.00 (23/23) a	1.00 (12/12) a	1.00 (25/25) a
	Dissemination (legs)	1.00 (25/25) a	1.00 (23/23) a	1.00 (12/12) a	1.00 (25/25) a
	Transmission (saliva)	0.24 (6/25) a	0.00 (0/23) b	0.33 (4/12) a	0.24 (6/25) a
	Vertical (positive ovaries)	1.00 (25/25) a	1.00 (23/23) a	1.00 (12/12) a	1.00 (25/25) a
	Mean (SE) Positive Ovaries	1.5 × 10^3^ pfu/mL a (±2.8 × 10^2^ pfu/mL)	2.2 × 10^3^ pfu/mL a (±5.2 × 10^2^ pfu/mL)	3.6 × 10^3^ pfu/mL a (±8.1 × 10^2^ pfu/mL)	3.2 × 10^3^ pfu/mL a (±1.4 × 10^3^ pfu/mL)
2	Infection (bodies)	NA	1.00 (13/13) a	1.00 (10/10) a	NA
	Dissemination (legs)	NA	1.00 (13/13) a	1.00 (10/10) a	NA
	Transmission (saliva)	NA	0.00 (0/13) a	0.20 (2/10) b	NA
	Vertical (positive ovaries)	NA	1.00 (13/13) a	1.00 (10/10) a	NA
	Mean (SE) Positive Ovaries	NA	8.3 × 10^2^ pfu/mL a (±3.2 × 10^2^ pfu/mL)	4.3 × 10^3^ pfu/mL a (±1.0 × 10^3^ pfu/mL)	NA
TOTALS:	Infection (bodies)	1.00 (25/25) a	1.00 (36/36) a	1.00 (22/22) a	1.00 (25/25) a
	Dissemination (legs)	1.00 (25/25) a	1.00 (36/36) a	1.00 (22/22) a	1.00 (25/25) a
	Transmission (saliva)	0.24 (6/25) a	0.00 (0/36) b	0.27 (6/22) a	0.24 (6/25) a
	Vertical (positive ovaries)	1.00 (25/25) a	1.00 (36/36) a	1.00 (22/22) a	1.00 (25/25) a
	Mean (SE) Positive Ovaries	1.5 × 10^3^ pfu/mL a (±2.8 × 10^2^ pfu/mL)	1.7 × 10^3^ pfu/mL a (±3.6 × 10^2^ pfu/mL)	3.9 × 10^3^ pfu/mL a (±6.3 × 10^2^ pfu/mL)	3.2 × 10^3^ pfu/mL a (±1.4 × 10^3^ pfu/mL)

NA indicates that data were not available for that transmission parameter.

**Table A2:** Connecticut *Aedes albopictus* LACV-oral feeding data by trial expressed as proportion infected out of total number tested. N values in parentheses. Values in parentheses in ovary titer rows indicate the standard error. Letters indicate significance (*p* < 0.05) between strains within each transmission parameter in each trial (ANOVA, Tukey’s multiple comparisons).

CT *Aedes albopictus* LACV Exposure by Oral Feeding					
LACV Strain (Lineage)					
Trial	Transmission Parameter	NC97 (I)	TX09 (II)	CT18 (III)	NY05 (III)
1	Infection (bodies)	0.33 (4/12) a	0.83 (5/6) b	1 (14/14) b	0.63 (5/8) ab
	Dissemination (legs)	0.08 (1/12) a	0.83 (5/6) bc	1 (14/14) c	0.50 (4/8) ab
	Transmission (saliva)	0.00 (0/12) a	0.50 (3/6) bc	0.64 (9/14) c	0.13 (1/8) ab
2	Infection (bodies)	0.73 (22/30) bc	0.97 (37/38) a	0.88 (35/40) ab	0.59 (24/41) c
	Dissemination (legs)	0.70 (21/30) bc	0.92 (35/38) a	0.88 (35/40) ab	0.54 (22/41) c
	Transmission (saliva)	0.07 (2/30) a	0.03 (1/38) a	0.05 (2/40) a	0.07 (3/41) a
3	Infection (bodies)	0.00 (0/17) a	0.30 (3/10) ab	0.40 (4/10) b	0.15 (2/13) ab
	Dissemination (legs)	0.00 (0/17) a	0.30 (3/10) a	0.30 (3/10) a	0.15 (2/13) a
	Transmission (saliva)	0.00 (0/17) a	0.20 (2/10) a	0.20 (2/10) a	0.08 (1/13) a
	Vertical (positive ovaries)	0.00 (0/17) a	0.30 (3/10) a	0.30 (3/10) a	0.15 (2/13) a
	Mean (SE) Positive Ovaries	0 pfu/mL a (±0 pfu/mL)	7.5 × 10^3^ pfu/mL a (±3.4 × 10^3^ pfu/mL)	7.7 × 10^2^ pfu/mL a (±5.0 × 10^2^ pfu/mL)	3.5 × 10^4^ pfu/m b (±2.3 × 10^4^ pfu/mL)
4	Infection (bodies)	0.00 (0/30) a	0.73 (22/30) b	0.60 (18/30) b	0.1 (3/30) a
	Dissemination (legs)	0.00 (0/30) a	0.63 (19/30) b	0.57 (17/30) b	0.10 (3/30) a
	Transmission (saliva)	0.00 (0/30) a	0.17 (5/30) a	0.10 (3/30) a	0.00 (0/30) a
	Vertical (positive ovaries)	0.00 (0/30) a	0.60 (18/30) b	0.50 (15/30) b	0.10 (3/30) a
	Mean (SE) Positive Ovaries	0 pfu/mL a (±0 pfu/mL)	1.7 × 10^3^ pfu/mL a (±4.0 × 10^2^ pfu/mL)	8.9 × 10^3^ pfu/mL b (±3.0 × 10^3^ pfu/mL)	2.5 × 10^4^ pfu/mL c (±1.2 × 10^4^ pfu/mL)
TOTALS:	Infection (bodies)	0.30 (27/89) a	0.80 (67/84) b	0.76 (71/94) b	0.35 (32/92) a
	Dissemination (legs)	0.25 (22/89) a	0.75 (62/84) b	0.73 (69/94) b	0.34 (31/92) a
	Transmission (saliva)	0.02 (2/89) a	0.13 (11/84) a	0.17 (16/94) a	0.05 (5/92) a
	Vertical (positive ovaries)	0.00 (0/30) a	0.53 (21/40) b	0.43 (17/40) b	0.16 (5/32) a
	Mean (SE) Positive Ovaries	0 pfu/mL a (±0 pfu/mL)	2.6 × 10^3^ pfu/mL a (±5.1 × 10^2^ pfu/mL)	6.8 × 10^3^ pfu/mL a (±7.1 × 10^2^ pfu/mL)	2.9 × 10^4^ pfu/mL a (±2.2 × 10^3^ pfu/mL)

**Table A3:** Connecticut *Aedes albopictus* LACV-inoculation data by trial expressed as proportion infected out of total number tested. N values in parentheses. Values in parentheses in ovary titer rows indicate the standard error. Letters indicate significance (*p* < 0.05) between strains within each transmission parameter in each trial (ANOVA, Tukey’s multiple comparisons).

CT *Aedes albopictus* LACV Exposure by Intrathoracic Inoculation					
		LACV Strain (Lineage)			
Trial	Transmission Parameter	NC97 (I)	TX09 (II)	CT18 (III)	NY05 (III)
1	Infection (bodies)	1.00 (30.30) a	1.00 (30.30) a	1.00 (24/24) a	1.00 (30.30) a
	Dissemination (legs)	1.00 (30/30) a	1.00 (30/30) a	1.00 (24/24) a	1.00 (30/30) a
	Transmission (saliva)	0.03 (1/30) a	0.10 (3/30) a	0.42 (10/24) b	0.13 (4/30) ab
	Vertical (positive ovaries)	0.97 (29/30) a	1.00 (30/30) a	1.00 (24/24) a	0.97 (29/30) a
	Mean (SE) Positive Ovaries	1.1 × 10^3^ pfu/mL a (±3.2 × 10^2^ pfu/mL)	2.6 × 10^3^ pfu/mL a (±5.0 × 10^2^ pfu/mL)	3.4 × 10^3^ pfu/mL a (±7.1 × 10^2^ pfu/mL)	9.0 × 10^3^ pfu/mL b (±2.3 × 10^3^ pfu/mL)

**Table A4:** Virginia *Aedes triseriatus* oral feeding data by trial expressed as proportion infected out of total number tested. N values in parentheses. Parentheses in the ovary titer rows indicate the standard error. Letters indicate significance (*p* < 0.05) between strains within each transmission parameter in each trial. (ANOVA, Tukey’s multiple comparisons).

VA *Aedes triseriatus* LACV Exposure by Oral Feeding					
Trial	Transmission Parameter	NC97	TX09	CT18	NY05
1	Infection (bodies)	0.38 (6/16) a	0.94 (17/18) b	0.95 (21/22) b	0.31 (5/16) a
	Dissemination (legs)	0.13 (2/16) a	0.72 (13/18) b	0.32 (7/22) a	0.19 (3/16) a
	Transmission (saliva)	0.00 (0/16) a	0.28 (5/18) a	0.23 (5/22) a	0.06 (1/16) a
2	Infection (bodies)	0.53 (8/15) a	1 (10/10) b	0.75 (9/12) ab	0.75 (6/8) ab
	Dissemination (legs)	0.00 (0/15) a	0.80 (8/10) b	0.67 (8/12) b	0.38 (3/8) ab
	Transmission (saliva)	0.00 (0/15) a	0.40 (4/10) ab	0.67 (8/12) b	0.13 (1/8) a
3	Infection (bodies)	0.57 (17/30) a	1.00 (30/30) b	1.00 (30/30) b	0.73 (22/30) a
	Dissemination (legs)	0.23 (7/30) a	0.90 (27/30) b	0.73 (22/30) b	0.67 (20/30) b
	Transmission (saliva)	0.00 (0/30) a	0.37 (10/27) b	0.30 (9/30) b	0.37 (11/30) b
	Vertical (positive ovaries)	0.43 (3/30) a	0.93 (27/30) b	0.70 (21/30) bc	0.85 (17/30) c
	Mean (SE) Positive Ovaries	3.3 × 10^4^ pfu/mL a (±1.8 × 10^4^ pfu/mL)	7.0 × 10^4^ pfu/mL ab (±1.1 × 10^4^ pfu/mL)	9.6 × 10^4^ pfu/mL b (±1.6 × 10^4^ pfu/mL)	9.5 × 10^4^ pfu/mL b (±1.9 × 10^4^ pfu/mL)
TOTALS:	Infection (bodies)	0.51 (31/61) a	0.98 (57/58) b	0.94 (60/64) b	0.61 (33/54) a
	Dissemination (legs)	0.15 (9/61) a	0.83 (48/58) b	0.58 (37/64) c	0.48 (26/54) c
	Transmission (saliva)	0.00 (0/61) a	0.33 (19/58) b	0.34 (22/64) b	0.24 (13/54) b
	Vertical (positive ovaries)	0.1 (3/30) a	0.93 (27/30) b	0.70 (21/30) bc	0.56 (17/30) c
	Mean (SE) Positive Ovaries	3.3 × 10^4^ pfu/mL a (±1.8 × 10^4^ pfu/mL)	7.0 × 10^4^ pfu/mL ab (±1.1 × 10^4^ pfu/mL)	9.6 × 10^4^ pfu/mL b (±1.6 × 10^4^ pfu/mL)	9.5 × 10^4^ pfu/mL b (±1.9 × 10^4^ pfu/mL)

**Table A5:** Virginia *Aedes triseriatus* LACV-inoculation data by trial expressed as proportion infected out of total number tested. N values in parentheses. Values in parentheses in ovary titer rows indicate the standard error. Letters indicate significance (*p* < 0.05) between strains within each transmission parameter in each trial (ANOVA, Tukey’s multiple comparisons).

VA *Aedes triseriatus* LACV Exposure by Intrathoracic Inoculation					
		LACV Strain (Lineage)			
Trial	Transmission Parameter	NC97 (I)	TX09 (II)	CT18 (III)	NY05 (III)
1	Infection (bodies)	1.00 (44/44) a	1.00 (48/48) a	1.00 (43/43) a	1.00 (52/52) a
	Dissemination (legs)	0.98 (43/44) a	1.00 (48/48) a	1.00 (43/43) a	1.00 (52/52) a
	Transmission (saliva)	0.18 (8/44) a	0.17 (8/48) a	0.74 (32/43) b	0.67 (35/52) b
2	Infection (bodies)	1.00 (16/16) a	1.00 (30/30) a	1.00 (30/30) a	1.00 (30/30) a
	Dissemination (legs)	1.00 (16/16) a	1.00 (30/30) a	1.00 (30/30) a	1.00 (30/30) a
	Transmission (saliva)	0.25 (4/16) bc	0.13 (4/30) c	0.33 (10/30) b	0.60 (18/30) a
	Vertical (positive ovaries)	0.50 (8/16) b	1.00 (30/30) a	1.00 (30/30) a	0.97 (29/30) a
	Mean (SE) Positive Ovaries	4.9 × 10^2^ pfu/mL a (±8.3 × 10^1^ pfu/mL)	7.9 × 10^2^ pfu/mL a (±1.3 × 10^2^ pfu/mL)	2.1 × 10^3^ pfu/mL a (±3.1 × 10^2^ pfu/mL)	1.2 × 10^3^ pfu/mL a (±3.0 × 10^2^ pfu/mL)
3	Infection (bodies)	1.00 (30/30) a	NA	1.00 (30/30) a	NA
	Dissemination (legs)	1.00 (30/30) a	NA	1.00 (30/30) a	NA
	Transmission (saliva)	0.23 (7/30) a	NA	0.40 (12/30) b	NA
	Vertical (positive ovaries)	1.00 (30/30) a	NA	1.00 (30/30) a	NA
	Mean (SE) Positive Ovaries	3.5 × 10^3^ pfu/mL a (±1.8 × 10^3^ pfu/mL)	NA	1.0 × 10^4^ pfu/mL a (±3.4 × 10^3^ pfu/mL)	NA
TOTALS:	Infection (bodies)	1.00 (90/90) a	1.00 (78/78) a	1.00 (103/103) a	1.00 (82/82) a
	Dissemination (legs)	0.99 (89/90) a	1.00 (78/78) a	1.00 (103/103) a	1.00 (82/82) a
	Transmission (saliva)	0.21 (19/90) a	0.15 (12/78) a	0.52 (54/103) b	0.65 (53/82) c
	Vertical (positive ovaries)	0.83 (38/46) a	1.00 (30/30) b	1.00 (60/60) b	0.97 (29/30) ab
	Mean (SE) Positive Ovaries	2.9 × 10^3^ pfu/mL ab (±1.3 × 10^3^ pfu/mL)	7.9 × 10^2^ pfu/mL b (±1.3 × 10^2^ pfu/mL)	6.2 × 10^3^ pfu/mL a (±1.8 × 10^3^ pfu/mL)	1.2 × 10^3^ pfu/mL b (±3.0 × 10^2^ pfu/mL)

NA indicates that data were not available for that transmission parameter.

**Table A6:** Virginia *Aedes albopictus* LACV-oral feeding data by trial expressed as proportion infected out of total number tested. N values in parentheses. Values in parentheses in ovary titer rows indicate the standard error. Letters indicate significance (*p* < 0.05) between strains within each transmission parameter in each trial (ANOVA, Tukey’s multiple comparisons).

VA *Aedes albopictus* LACV Exposure by Oral Feeding					
		LACV Strain (Lineage)			
Trial	Transmission Parameter	NC97 (I)	TX09 (II)	CT18 (III)	NY05 (III)
1	Infection (bodies)	0.32 (12/37) a	1 (31/31) b	1 (31/31) b	0.79 (11/14) b
	Dissemination (legs)	0.24 (9/37) a	1 (31/31) b	1 (31/31) b	0.64 (9/14) c
	Transmission (saliva)	0.03 (1/37) a	0.06 (2/31) a	0.39 (12/31) b	0.36 (5/14) b
2	Infection (bodies)	0.12 (8/69) a	0.98 (53/54) b	0.92 (34/37) b	0.13 (6/48) a
	Dissemination (legs)	0.03 (2/69) a	0.94 (51/54) b	0.76 (28/37) c	0.08 (4/48) a
	Transmission (saliva)	0.01 (1/69) a	0.06 (3/54) ab	0.16 (6/37) b	0 (0/48) a
3	Infection (bodies)	0.40 (12/30) b	NA	0.67 (20/30) a	0.30 (9/30) b
	Dissemination (legs)	0.33 (10/30) b	NA	0.60 (18/30) a	0.30 (9/30) b
	Transmission (saliva)	0.00 (0/30) a	NA	0.17 (5/30) a	0.07 (2/30) a
	Vertical (positive ovaries)	0.27 (8/30) b	NA	0.60 (18/30) a	0.30 (9/30) b
	Mean (SE) Positive Ovaries	1.2 × 10^4^ pfu/mL ab (±1.2 × 10^4^ pfu/mL)	NA	5.5 × 10^4^ pfu/mL a (±1.1 × 10^4^ pfu/mL)	7.3 × 10^3^ pfu/mL b (±4.3 × 10^3^ pfu/mL)
4	Infection (bodies)	NA	0.53 (16/30) a	NA	0.07 (2/30) b
	Dissemination (legs)	NA	0.47 (14/30) a	NA	0.07 (2/30) b
	Transmission (saliva)	NA	0.07 (2/30) a	NA	0.00 (0/30) a
	Vertical (positive ovaries)	NA	0.43 (13/30) a	NA	0.07 (2/30) b
	Mean (SE) Positive Ovaries	NA	7.0 × 10^3^ pfu/mL a (±1.5 × 10^3^ pfu/mL)	NA	2.2 × 10^4^ pfu/mL a (±1.2 × 10^4^ pfu/mL)
TOTAL:	Infection (bodies)	0.24 (32/136) a	0.87 (100/115) b	0.87 (85/98) b	0.21 (26/122) a
	Dissemination (legs)	0.15 (21/136) a	0.83 (96/115) b	0.79 (77/98) b	0.20 (24/122) a
	Transmission (saliva)	0.01 (2/136) a	0.06 (7/115) a	0.23 (23/98) b	0.06 (7/122) a
	Vertical (positive ovaries)	0.27 (8/30) bc	0.43 (13/30) ab	0.60 (18/30) a	0.18 (11/60) c
	Mean (SE) Positive Ovaries	1.2 × 10^4^ pfu/mL ab (±1.2 × 10^4^ pfu/mL)	7.0 × 10^3^ pfu/mL a (±1.5 × 10^3^ pfu/mL)	5.5 × 10^4^ pfu/mL b (±1.1 × 10^4^ pfu/mL)	1.0 × 10^4^ pfu/mL a (±4.3 × 10^3^ pfu/mL)

NA indicates that data were not available for that transmission parameter.

**Table A7:** VA *Aedes albopictus* LACV-inoculation data by trial expressed as proportion infected out of total number tested. N values in parentheses. Values in parentheses in ovary titer rows indicate the standard error. Letters indicate significance (*p* < 0.05) between strains within each transmission parameter in each trial (ANOVA, Tukey’s multiple comparisons).

VA *Aedes albopictus* LACV Exposure by Intrathoracic Inoculation					
		LACV Strain (lineage)			
Trial	Transmission Parameter	NC97 (I)	TX09 (II)	CT18 (III)	NY05 (III)
1	Infection (bodies)	1.00 (30/30) a	1.00 (30/30) a	1.00 (30/30) a	1.00 (30/30) a
	Dissemination (legs)	1.00 (30/30) a	1.00 (30/30) a	1.00 (30/30) a	1.00 (30/30) a
	Transmission (saliva)	0.13 (4/30) ab	0.10 (3/30) a	0.30 (9/30) c	0.27 (8/30) bc
	Vertical (positive ovaries)	0.87 (26/30) a	0.73 (22/30) a	1.00 (30/30) a	1.00 (30/30) a
	Mean (SE) Positive Ovaries	1.2 × 10^3^ pfu/mL a (±3.1 × 10^2^ pfu/mL)	7.6 × 10^2^ pfu/mL a (±2.5 × 10^2^ pfu/mL)	2.9 × 10^3^ pfu/mL a (±1.5 × 10^3^ pfu/mL)	1.2 × 10^3^ pfu/mL a (±5.9 × 10^2^ pfu/mL)
